# CYP2D6 Metabolizer Phenotype and New Persistent Opioid Use After Spine Surgery

**DOI:** 10.1177/21925682261495118

**Published:** 2026-09-30

**Authors:** Samer G. Salman, Rohan A. Phadke, Krishna K. Anand, Joseph W. Guarnieri, Ethan Waisberg, James Rizkalla, Nathan J. Lee

**Affiliations:** 1 School of Medicine, 3989 Baylor College of Medicine, Houston, TX, USA; 2 Department of Orthopaedic Surgery, 12273 University of Missouri-Kansas City, Kansas City, MO, USA; 3 Guarnieri Research Group, Philadelphia, PA, USA; 4 824783 Blue Marble Space Institute of Science, Seattle, WA, USA; 5 Department of Medical Genetics, 2152 University of Cambridge, Cambridge, UK; 6 Department of Orthopaedic Surgery, 22683 Baylor University Medical Center, Dallas, TX, USA; 7 Midwest Orthopaedics at Rush, Rush University Medical Center, Chicago, IL, USA

**Keywords:** CYP2D6, pharmacogenomics, spine surgery, opioid use, persistent opioid use, postoperative pain, precision medicine, hydrocodone, genetic polymorphism, All of Us Research Program

## Abstract

**Study Design:**

Retrospective Cohort Study.

**Objectives:**

To determine whether cytochrome P450 2D6 (CYP2D6) metabolizer phenotype is associated with new persistent opioid use after cervical, thoracic, or lumbar spine surgery among opioid-naive adults.

**Methods:**

Adults with whole-genome sequencing data who underwent cervical, thoracic, or lumbar fusion or decompression between May 2018 and January 1, 2025, were identified in the All of Us Research Program. Participants had 365 days of baseline observation, 180 days of postoperative follow-up, and no opioid fills from 365 to 30 days before surgery. CYP2D6 phenotype was categorized as poor, intermediate, normal, or ultrarapid metabolizer. New persistent opioid use required a perioperative opioid fill and another fill 90 to 180 days postoperatively. Associations were estimated using Firth-penalized logistic regression adjusted for ancestry principal components and clinical covariates.

**Results:**

Among 3,820 participants, 460 (12.0%) developed new persistent opioid use. The overall association across CYP2D6 phenotypes was significant (χ^2^ = 15.30; *P* = .002). Compared with normal metabolizers, adjusted odds ratios were 1.48 (95% CI, 1.00–2.18) for poor, 1.05 (95% CI, 0.85–1.30) for intermediate, and 1.50 (95% CI, 0.89–2.54) for ultrarapid metabolizers. Poor metabolizers receiving hydrocodone had higher odds of persistent use (OR, 2.15; 95% CI, 1.07–4.32).

**Conclusions:**

CYP2D6 phenotype was associated with new persistent opioid use after spine surgery, with higher risk estimates at the metabolic extremes. Extreme-phenotype estimates were reportable only in European-ancestry participants, so risk in other ancestry groups remains unknown. Medication-specific replication in larger, ancestry-diverse cohorts is needed before clinical implementation.

## Introduction

Opioid treatment initiated after surgery may extend beyond the expected period of acute recovery among patients without prior opioid exposure. New persistent opioid use, commonly defined as opioid prescription fulfillment 90 to 180 days after surgery among previously opioid-naive patients, occurs in approximately 6% to 7% of surgical patients.^1,2^ Spine surgery is an important setting for this outcome because postoperative pain can be prolonged and opioid exposure is common.^3^ Persistent opioid use may affect recovery, patient counseling, postoperative surveillance, and opioid stewardship, making identification of susceptible patients clinically relevant.^4^

Established predictors of prolonged postoperative opioid use include preoperative opioid exposure, depression, anxiety, substance-use disorders, tobacco use, chronic pain conditions, procedural characteristics, and postoperative prescribing patterns.^3,5-8^ Preoperative opioid use is consistently among the strongest predictors after lumbar fusion.^3,7^ However, these clinical and behavioral factors do not fully explain why some opioid-naive patients continue using prescribed opioids months after surgery. Genetically determined variation in opioid metabolism may represent an additional source of susceptibility.

Cytochrome P450 2D6 (CYP2D6) bioactivates codeine and tramadol and contributes to metabolism of hydrocodone and oxycodone, although the clinical importance of CYP2D6 varies by opioid.^9,10^ Genetic variation produces poor, intermediate, normal, and ultrarapid metabolizer phenotypes, which can alter exposure to active opioid metabolites and potentially influence analgesic response, tolerability, and subsequent treatment.^9,11^ The Clinical Pharmacogenetics Implementation Consortium provides CYP2D6-based prescribing recommendations for codeine and tramadol, whereas evidence supporting genotype-guided prescribing of hydrocodone and oxycodone remains limited.^9^

Clinical evidence linking CYP2D6 phenotype to opioid-related outcomes has been inconsistent. Pharmacogenetically guided treatment has improved pain outcomes in some chronic-pain populations, particularly among patients receiving codeine or tramadol,^12^ and reduced CYP2D6 activity has been associated with greater pain-related healthcare utilization.^13^ Conversely, postoperative studies have found no improvement in acute pain or opioid consumption with CYP2D6-guided prescribing and no consistent association between metabolizer phenotype and short-term outcomes.^10,14^ Whether CYP2D6 variation is associated with opioid use extending beyond acute postoperative recovery remains unknown.

We evaluated the association between CYP2D6 metabolizer phenotype and new persistent opioid use among opioid-naive adults undergoing cervical, thoracic, or lumbar fusion or decompression in the All of Us Research Program.^15,16^ We hypothesized a nonlinear association in which poor and ultrarapid metabolizers would have a higher risk of new persistent opioid use than normal metabolizers, while intermediate metabolizers would have a similar risk.

## Methods

### Data source and setting

We conducted this study within the All of Us Research Program using Curated Data Repository version 9 (C2025Q4R6; data cutoff, January 1, 2025), mapped to the Observational Medical Outcomes Partnership Common Data Model version 5.3. All analyses were performed within the All of Us Researcher Workbench as secondary analyses of de-identified data. In accordance with All of Us disclosure requirements, counts were rounded to the nearest 20, cells containing 1 to 20 participants were suppressed, and no participant-level data were exported.

### Cohort

We identified adults undergoing an index cervical, thoracic, or lumbar fusion or decompression who had whole-genome sequencing data and were opioid-naive before surgery. Opioid-naive status was defined as no opioid fills from 365 to 30 days before the index procedure. Participants were required to have at least 365 days of baseline observation and 180 days of postoperative follow-up. The index date was the first qualifying spine procedure, and patients were classified into fusion, decompression, or combined-procedure strata. We also assigned each patient an anatomic region from the set of levels recorded across all qualifying procedures, ignoring level-agnostic add-on and instrumentation codes. A single level defined the region as cervical, thoracic, or lumbar, more than one as multilevel (procedures spanning more than one spinal region), and none as unassignable; thoracic, multilevel, and unassignable were grouped as other. Among 3,920 patients with an available CYP2D6 call, we excluded those with indeterminate calls, yielding a primary analytic cohort of 3,820 patients.

### Exposure

The primary exposure was CYP2D6 metabolizer phenotype derived from precomputed Cyrius star-allele calls. CYP2D6 contributes to the bioactivation of codeine and tramadol and the metabolism of hydrocodone and oxycodone. Phenotypes were categorized as poor, intermediate, normal, or ultrarapid metabolizer, reflecting genetically determined CYP2D6 activity. Normal metabolizers served as the reference group. Ten genetic ancestry principal components were included to account for population structure.

### Outcome

The primary outcome was new persistent opioid use, defined according to Brummett 2017. Participants were required to have both a perioperative opioid fill from 7 days before through 14 days after surgery and at least one additional opioid fill from 90 through 180 days after surgery. Because all participants were opioid-naive at baseline, this definition captured opioid use initiated around surgery and persisting for several months. Cohort construction, CYP2D6 phenotype assignment, the perioperative timeline, and the outcome definition are summarized in Figure 1.

**Figure 1.**
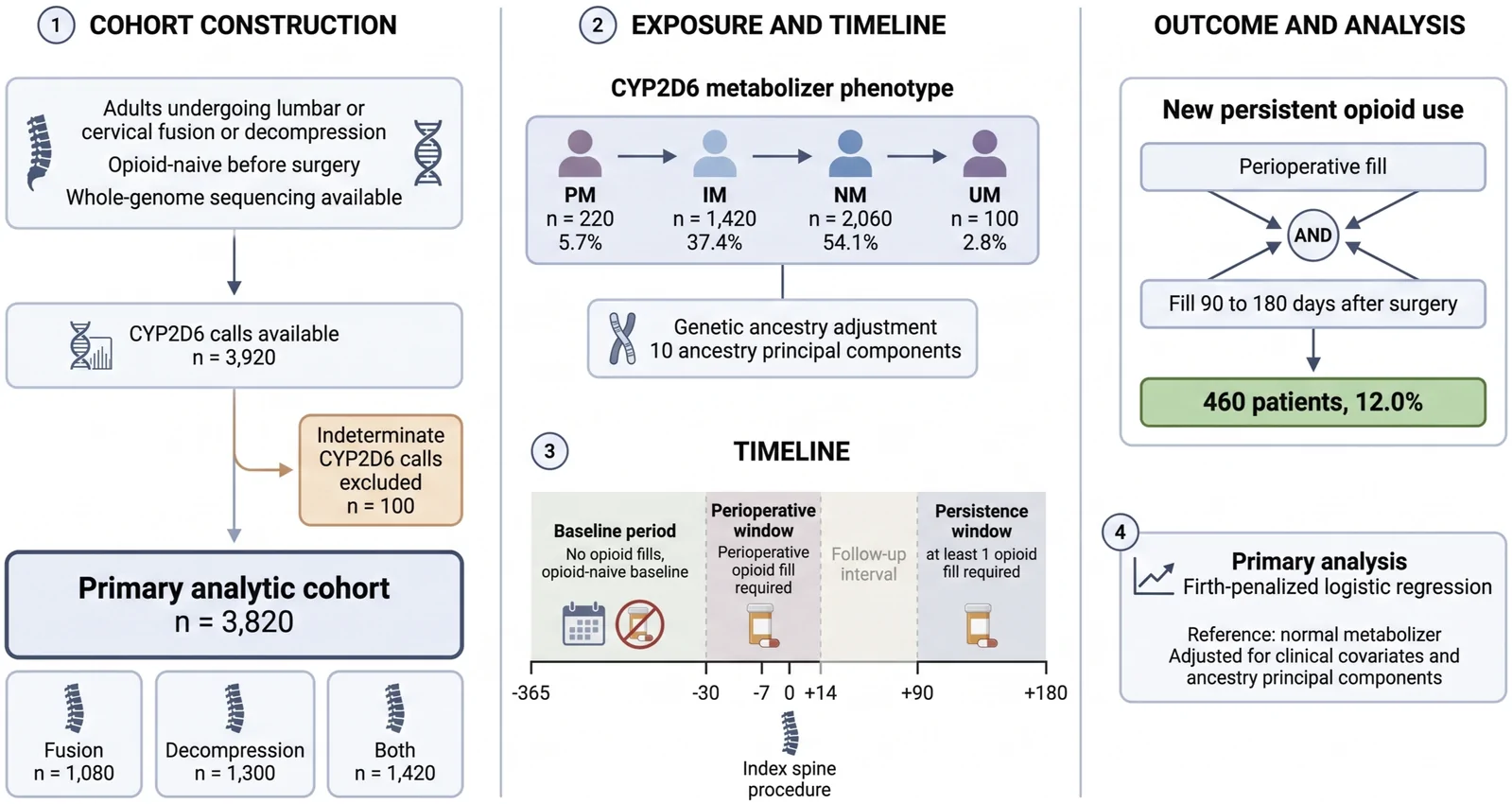
Study design, cohort construction, and definition of new persistent opioid use. Opioid-naive adults undergoing cervical, thoracic, or lumbar fusion or decompression were identified within the All of Us Research Program. Among 3,920 patients with CYP2D6 calls, 100 indeterminate calls were excluded, leaving 3,820 patients in the primary analytic cohort. New persistent opioid use required both a perioperative opioid fill from 7 days before through 14 days after surgery and at least one additional fill from 90 through 180 days after surgery. Associations were estimated using Firth-penalized logistic regression adjusted for clinical covariates and 10 ancestry principal components

### Statistical analysis

We estimated associations between the four-level CYP2D6 phenotype and new persistent opioid use using Firth-penalized logistic regression. The primary model adjusted for 10 ancestry principal components, age, sex, pre-index body mass index, procedure stratum, baseline back pain, depression, anxiety, substance-use disorder, and Charlson comorbidity index. Phenotypes were modeled categorically without collapsing groups or imposing a linear trend because the hypothesized association was U-shaped, with potentially increased risk among poor and ultrarapid metabolizers through different mechanisms and an approximately null association among intermediate metabolizers.

We prespecified two key secondary analyses. First, we assessed whether associations followed a substrate-dependence gradient across perioperative opioid tiers, ranging from T3 codeine or tramadol exposure to T0 non-substrate exposure. Second, we evaluated within-ancestry covariate balance and fitted a sensitivity model incorporating baseline healthcare-use measures. Because genotype is fixed at conception, genetic ancestry was treated as the principal potential confounder; healthcare-use measures were used primarily as balance and residual-bias diagnostics rather than primary-model covariates. We additionally evaluated a PPI-naive negative-control outcome. We refitted the primary Firth model within each anatomic region and each procedure stratum, retaining procedure stratum as a covariate in the region models and dropping it in the procedure models. We also tested phenotype-by-region and phenotype-by-procedure-type interactions in the full cohort by penalized likelihood-ratio test. These stratified analyses were exploratory and were not prespecified. Analyses were conducted in Python using statsmodels, a self-contained Firth regression routine, lifelines, scikit-learn, and Hail.

## Results

### Cohort and baseline characteristics

Among 3,920 patients with a CYP2D6 call, 3,820 remained after exclusion of indeterminate calls and formed the primary analytic cohort. New persistent opioid use occurred in 460 patients (12.0%). The cohort included 220 poor metabolizers (PMs; 5.7%), 1,420 intermediate metabolizers (IMs; 37.4%), 2,060 normal metabolizers (NMs; 54.1%), and 100 ultrarapid metabolizers (UMs; 2.8%). Most patients were of European ancestry (n=2,840; 74.3%), followed by African (n=480; 12.7%), Admixed American (n=420; 10.9%), and East Asian (n=60; 1.4%) ancestry. Only the European stratum contained reportable PM and UM cells.

Mean age was 59.9 years (SD 13.7), 1,940 patients (50.9%) were women, and mean BMI was 30.2 (SD 6.4). Procedures included fusion alone in 1,080 patients (28.4%), decompression alone in 1,300 (34.2%), and both in 1,420 (37.3%); the operative region was cervical in 960 (25.4%), lumbar in 2,280 (59.9%), and other in 560 (14.7%). Overall, 2,800 patients (73.1%) filled a perioperative opioid and 360 (9.4%) filled a CYP2D6-dependent prodrug, codeine or tramadol. Baseline demographic, clinical, procedural, and outcome characteristics overall and by CYP2D6 phenotype are summarized in Table 1.

**Table 1.** Baseline characteristics of the analytic cohort, overall and by CYP2D6 metabolizer phenotype

Characteristic	Overall (N=3,820)	PM (n=220)	IM (n=1,420)	NM (n=2,060)	UM (n=100)
Age, years, mean ± SD	59.9 ± 13.7	62.0 ± 13.4	59.6 ± 13.8	59.8 ± 13.6	61.2 ± 13.1
Female, n (%)	1,940 (50.9%)	120 (50.9%)	740 (52.5%)	1,020 (49.8%)	60 (50.5%)
BMI, mean ± SD	30.2 ± 6.4	30.1 ± 7.3	30.3 ± 6.8	30.1 ± 6.1	29.7 ± 5.5
Fusion, n (%)	1,080 (28.4%)	80 (32.6%)	420 (29.0%)	580 (27.7%)	40 (28.0%)
Decompression, n (%)	1,300 (34.2%)	80 (32.6%)	500 (35.4%)	700 (33.5%)	40 (36.4%)
Both, n (%)	1,420 (37.3%)	80 (34.9%)	500 (35.6%)	800 (38.9%)	40 (35.5%)
Cervical, n (%)	960 (25.4%)	60 (27.3%)	360 (25.4%)	520 (25.2%)	20 (20.0%)
Lumbar, n (%)	2,280 (59.9%)	120 (54.5%)	840 (59.2%)	1,240 (60.2%)	80 (80.0%)
Other, n (%)	560 (14.7%)	40 (18.2%)	220 (15.5%)	300 (14.6%)	≤20
Baseline back pain, n (%)	2,740 (71.7%)	160 (69.7%)	1,020 (71.9%)	1,480 (71.3%)	80 (79.4%)
Depression, n (%)	1,020 (26.5%)	40 (21.6%)	400 (27.3%)	540 (26.2%)	40 (30.8%)
Anxiety, n (%)	940 (24.7%)	40 (22.0%)	360 (24.6%)	520 (25.3%)	20 (21.5%)
Substance or opioid use disorder, n (%)	440 (11.7%)	20 (11.9%)	160 (11.8%)	240 (11.2%)	≤20
Tobacco, n (%)	480 (12.6%)	20 (11.9%)	180 (12.9%)	260 (12.3%)	≤20
Comorbidity count, mean	1.05	0.95	1.06	1.04	1.21
New persistent opioid use, n (%)	460 (12.0%)	40 (15.6%)	160 (11.9%)	240 (11.5%)	≤20

Abbreviations: BMI, body mass index; CYP2D6, cytochrome P450 2D6; IM, intermediate metabolizer; NM, normal metabolizer; PM, poor metabolizer; SD, standard deviation; UM, ultrarapid metabolizer. Counts are rounded to the nearest 20, and cells with 20 or fewer participants are suppressed in accordance with All of Us Research Program disclosure requirements. Percentages were calculated before rounding and therefore may not correspond exactly to the reported counts or sum to 100%. Other anatomic region comprises thoracic, multilevel (procedures spanning more than one spinal region), and unassignable operative levels. Anatomic-region percentages within each phenotype column were calculated from the rounded counts shown, because pre-rounding values were not available for those cells. Comorbidity count is a tally of seven recorded comorbidity conditions and is not the Charlson comorbidity index used as a covariate in the regression models, whose cohort mean was 1.10. The suppressed cells are all in the ultrarapid metabolizer column: other anatomic region, substance or opioid use disorder, tobacco, and new persistent opioid use.

PM indicates poor metabolizer; IM, intermediate metabolizer; NM, normal metabolizer; UM, ultrarapid metabolizer; and BMI, body mass index. Counts are rounded to the nearest 20, and cells of 20 or fewer are suppressed per All of Us disclosure policy. Percentages were calculated before rounding and therefore may not sum exactly to the reported group totals. That statement does not apply to the anatomic-region percentages within each phenotype column, which were derived from the rounded counts because pre-rounding values were unavailable. Other anatomic region comprises thoracic, multilevel (procedures spanning more than one spinal region), and unassignable operative levels. UM cells for other anatomic region, substance or opioid use disorder, tobacco use, and new persistent opioid use were suppressed.

### Primary association

Relative to NMs, PMs had higher estimated odds of new persistent opioid use (OR 1.48, 95% CI 1.00–2.18; *P*= 0.052), as did UMs (OR 1.50, 95% CI 0.89–2.54; *P*= 0.13), whereas IMs remained near the null (OR 1.05, 95% CI 0.85–1.30; *P*= 0.64). Although no individual contrast crossed the conventional significance threshold, the joint 3-degree-of-freedom penalized likelihood-ratio test supported an overall association between the 4-level CYP2D6 phenotype and the outcome (χ^2^=15.30; *P*= 0.0016). Complete adjusted estimates and the joint phenotype test are reported in Table 2. Figure 2 displays the categorical pattern, with higher point estimates at the PM and UM extremes and an IM estimate near the NM reference. Results were materially unchanged when adjustment included 0, 5, 10, or 16 ancestry principal components.

**Table 2.** CYP2D6 metabolizer phenotype and new persistent opioid use

CYP2D6 phenotype	Odds ratio	95% CI	*p*-*value*
Normal metabolizer (reference)	1.00 [Reference]	​	​
Intermediate metabolizer	1.05	0.85–1.30	0.64
Poor metabolizer	1.48	1.00–2.18	0.052
Ultrarapid metabolizer	1.50	0.89–2.54	0.13
Joint 3-degree-of-freedom penalized likelihood-ratio test for CYP2D6 phenotype: χ^2^ = 15.30; P = 0.0016			

Abbreviations: CI, confidence interval; CYP2D6, cytochrome P450 2D6. Odds ratios were estimated using Firth-penalized logistic regression, with normal metabolizers as the reference group. The model adjusted for age, sex, pre-index body mass index, procedure stratum, baseline back pain, depression, anxiety, substance or opioid use disorder, Charlson comorbidity index, and ancestry principal components 1 through 10. Estimates stratified by anatomic region and procedure type are reported in eTable 2 and eFigure 1 in the Supplement.

**Figure 2.**
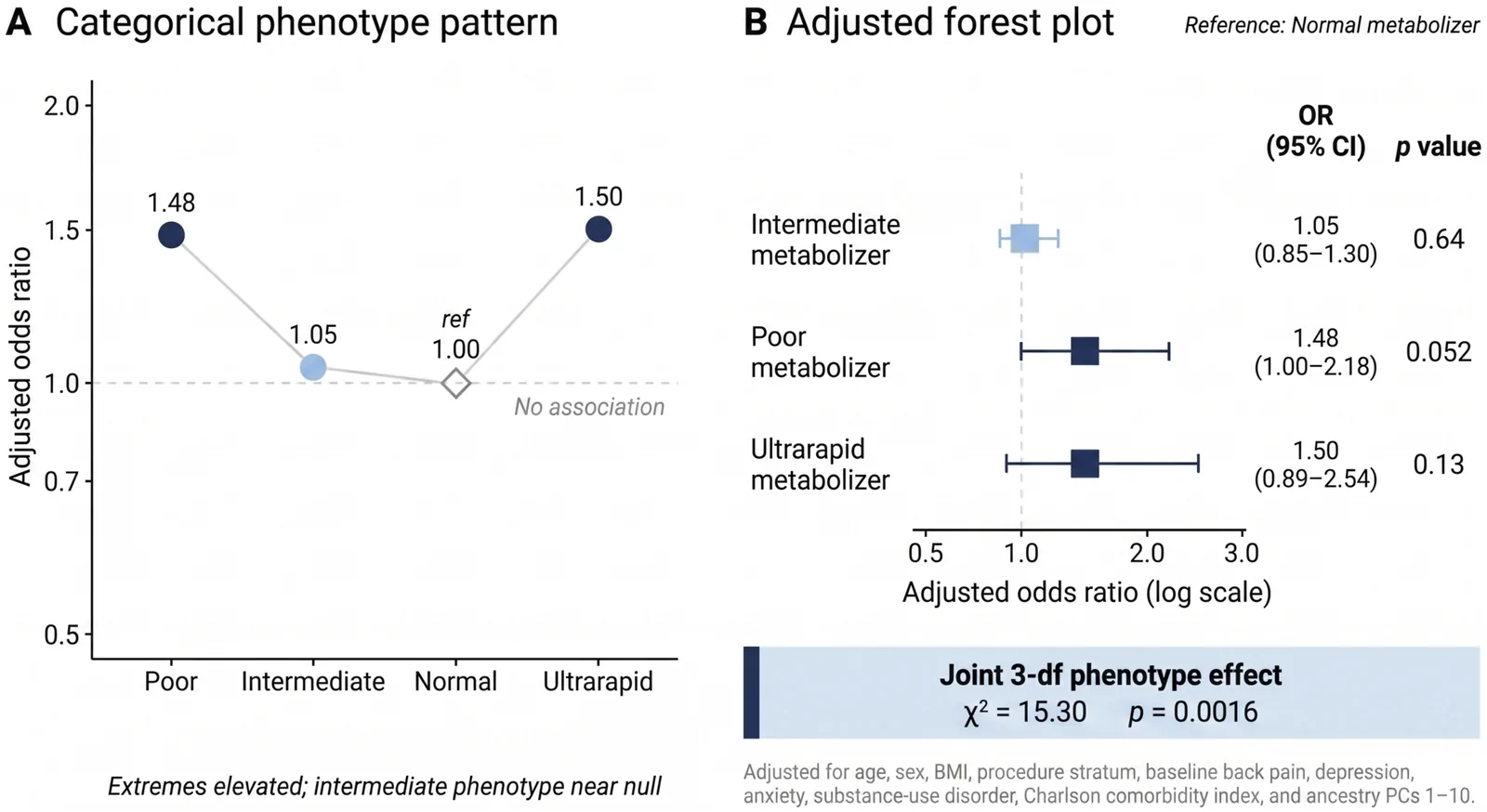
Association between CYP2D6 metabolizer phenotype and new persistent opioid use after spine surgery. Panel A displays the categorical pattern of adjusted odds ratios across increasing CYP2D6 metabolizer function, with elevated estimates at the poor- and ultrarapid-metabolizer extremes and an intermediate-metabolizer estimate near the normal-metabolizer reference. Panel B presents phenotype-specific estimates from the Firth-penalized logistic regression. The joint 3-degree-of-freedom phenotype effect was significant (χ^2^=15.30; *P*= 0.0016), although the individual poor- and ultrarapid-metabolizer contrasts were imprecisely estimated

The primary model adjusted for age, sex, pre-index BMI, procedure stratum, baseline back pain, depression, anxiety, substance-use disorder, Charlson comorbidity index, and principal components 1 through 10.

### Stratified analyses by anatomic region and procedure type

The primary model was refitted within each anatomic region and each procedure stratum (eTable 2 and eFigure 1 in Supplement). Only 80 patients (1.9%) had a thoracic-only procedure. Event rates were 9.9% in the cervical group and 10.8% in the lumbar group, against 21.1% in the other group, roughly double the cervical and lumbar rates. Multilevel procedures, meaning surgery involving more than one spinal region, accounted for 400 of those 560 patients, and unassignable levels for 80. Surgery across more than one region is therefore a likely explanation for the higher rate, but that explanation was not tested. Poor-metabolizer odds ratios exceeded 1.0 in all six strata, ranging from 1.05 to 1.77. The joint 3-degree-of-freedom test was nominally significant in four of the six strata, including the two largest (lumbar, *P* = 0.004; combined procedures, *P* = 0.006). Ultrarapid-metabolizer estimates were inconsistent in direction, 2.17 in combined procedures against 0.89 in decompression, each based on 40 ultrarapid metabolizers. The ultrarapid-metabolizer contrast in the other-region stratum could not be estimated because of thin cells. In the full cohort, the phenotype-by-region interaction was not significant (χ^2^ = 12.04; df = 6; *P* = 0.061), whereas the phenotype-by-procedure-type interaction was nominally significant (χ^2^ = 13.69; df = 6; *P* = 0.033). Neither test was adjusted for the two tests performed, and neither was prespecified.

### Substrate-specific analyses

To assess whether the association strengthened with greater CYP2D6 dependence, we examined four perioperative opioid substrate tiers (eTable 1 in Supplement). Among PMs, the OR was 1.28 (95% CI 0.44–3.72; *P*= 0.65) for T3 prodrugs, codeine or tramadol; 2.15 (95% CI 1.07–4.32; *P*= 0.032) for T2 hydrocodone; and 1.08 (95% CI 0.58–2.02; *P*= 0.80) for T1 oxycodone. The T0 non-substrate tier could not be estimated because of sparse cells. The association was therefore concentrated in the hydrocodone tier rather than increasing monotonically with CYP2D6 dependence. Neither the ordinal-tier trend (OR 1.089, 95% CI 0.958–1.237; *P*= 0.19) nor the PM-or-UM-by-tier interaction (OR 1.073, 95% CI 0.718–1.602; *P*= 0.73) was significant. Counts are rounded to the nearest 20 per All of Us disclosure policy.

### Within-ancestry balance and healthcare-use sensitivity analysis

Within the European-ancestry stratum, PMs were generally balanced across measured baseline characteristics; the largest standardized mean difference was 0.155 for baseline proton-pump-inhibitor (PPI) use. UMs showed greater imbalance for baseline PPI use (standardized mean difference 0.262), and estimates were imprecise because only 60 UMs were available. Adding baseline healthcare-use measures attenuated the PM OR from 1.48 to 1.33, a 27.6% reduction on the log-odds scale, and the UM OR from 1.50 to 1.37, a 21.8% reduction. Residual associations of approximately 1.3 to 1.4 remained.

### Negative-control outcome

We evaluated new persistent PPI use among 3,300 patients without baseline PPI fills, using the same perioperative exposure window and 90- to 180-day persistence window as the primary analysis. Sixty events occurred. The PM OR was 1.88 (95% CI 0.77–4.61; *P*= 0.17), which was not statistically significant. The standard error of the log odds was 0.457, against 0.200 for the primary outcome, so the negative control was 2.3 times less precise. It had 80% power only for an odds ratio at or above approximately 3.6, far above the odds ratio near 1.5 observed for the primary outcome. The negative-control analysis was therefore an underpowered check and could not exclude residual confounding of the magnitude relevant to the primary estimate.

### Absolute risk, time-to-event, and prediction analyses

At the observed 12.0% baseline risk, the estimated absolute risks were 16.8% for PMs and 17.1% for UMs, corresponding to absolute differences of 4.8 and 5.0 percentage points, respectively. These differences are equivalent to approximately one additional event per 21 PMs and per 20 UMs. E-values were 2.31 for PMs and 2.37 for UMs. An aligned Cox model produced similar estimates for PMs (HR 1.30; *P*= 0.038) and UMs (HR 1.42; *P*= 0.041). The cross-validated clinical prediction model achieved an AUROC of 0.571 (SD 0.028), close to chance. Adding genotype gave an AUROC of 0.572 (SD 0.026), a ΔAUROC of +0.001. The substrate-specific, healthcare-use, negative-control, absolute-risk, and incremental-prediction findings are integrated in Figure 3.

**Figure 3.**
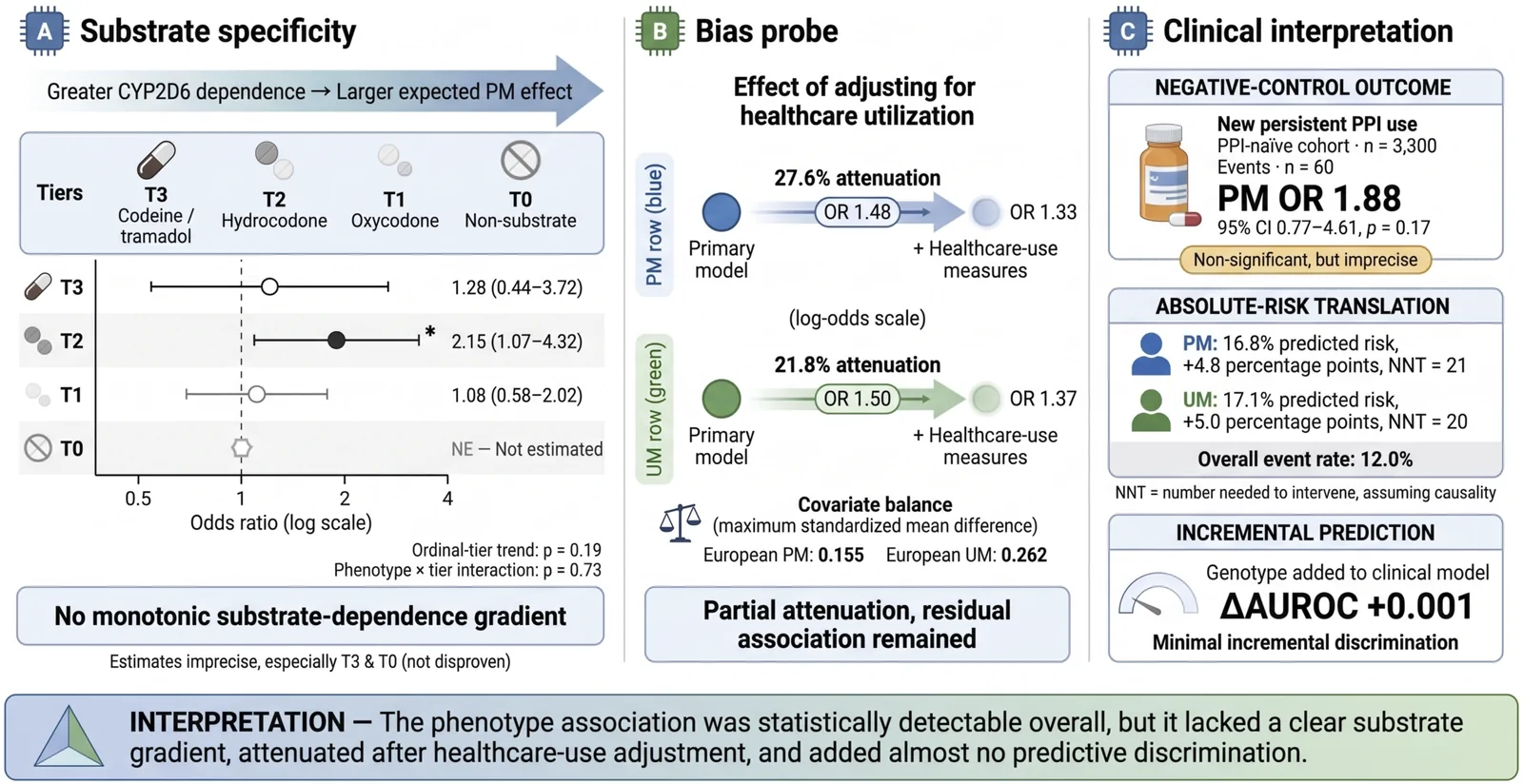
Triangulation of pharmacologic specificity, potential residual bias, and clinical utility. Panel A shows poor-metabolizer associations across perioperative opioid substrate tiers. The largest estimate occurred for hydrocodone rather than the strongest CYP2D6-dependent prodrugs, and neither the ordinal-tier trend nor the phenotype-by-tier interaction was significant. Panel B shows attenuation of the poor- and ultrarapid-metabolizer estimates after addition of baseline healthcare-use measures. Panel C summarizes the PPI-naive negative-control analysis, absolute-risk translations, and the negligible change in discrimination after CYP2D6 phenotype was added to the clinical prediction model. Absolute-risk and number-needed-to-treat estimates assume that the observed associations are causal and should therefore be interpreted cautiously

## Discussion

In this cohort of opioid-naive adults undergoing cervical, thoracic, or lumbar spine surgery, CYP2D6 metabolizer phenotype was associated with new persistent opioid use in the global four-category analysis (χ^2^=15.30; *P*= 0.0016). The pattern was nonlinear: poor metabolizers (OR, 1.48; 95% CI, 1.00–2.18) and ultrarapid metabolizers (OR, 1.50; 95% CI, 0.89–2.54) had higher point estimates than normal metabolizers, whereas intermediate metabolizers had a near-null estimate (OR, 1.05; 95% CI, 0.85–1.30). At the observed 12.0% baseline risk, the estimated absolute risk increase at either metabolic extreme was approximately 5 percentage points, equivalent to roughly one additional persistent-use event per 20 to 21 patients. These findings identify CYP2D6 phenotype as a candidate inherited susceptibility factor. The individual poor- and ultrarapid-metabolizer estimates remained imprecise, and the absolute-risk estimates require external validation before they can inform care.

The observed 12.0% incidence confirms that persistent opioid use is a clinically relevant outcome after spine surgery. Brummett et al. reported rates of 5.9% to 6.5% among opioid-naive adults undergoing diverse procedures using the same 90- to 180-day definition, while a recent meta-analysis estimated a pooled incidence of 7.15% overall, rising to 8.29% among studies applying the same 90- to 180-day definition.^1,2^ Spine-specific estimates vary because of differences in prior opioid exposure, procedure type, follow-up, and outcome definition.^3,4,17,18^ The present rate likely reflects the inclusion of fusion and decompression procedures, the perioperative fill requirement, and characteristics of the All of Us population. Persistent fills beyond the expected acute recovery period remain an important target for counseling, surveillance, and opioid stewardship.^6,19^

The principal contribution of this study is the extension of CYP2D6 pharmacogenomic evaluation from acute analgesic response to persistent opioid prescription use months after surgery. Prior studies have linked reduced CYP2D6 activity to poorer pain-related outcomes among patients receiving codeine, tramadol, or hydrocodone. Smith et al. reported improved pain control with CYP2D6-guided chronic pain management, particularly among intermediate and poor metabolizers receiving these agents.^12^ Nahid et al. found more pain-related emergency department visits among intermediate and poor metabolizers prescribed hydrocodone, tramadol, or codeine, with no comparable association for oxycodone.^13^ In the present study, the hydrocodone-specific estimate among poor metabolizers was elevated (OR, 2.15; 95% CI, 1.07–4.32), providing a focused hypothesis for replication.

Evidence from acute postoperative settings has been less consistent. The ADOPT PGx randomized trial showed that CYP2D6-guided prescribing altered opioid selection without improving acute pain or opioid use, and a secondary analysis found no phenotype association with 10-day pain or consumption.^10,14^ Differences in outcome, follow-up, surgical population, multimodal analgesia, and phenotype definition may explain the divergence. Persistent prescription use at 90 to 180 days reflects a broader process involving pain persistence, refill behavior, clinician prescribing, healthcare contact, adverse effects, and medication switching. CYP2D6 may therefore contribute to a longer postoperative prescribing trajectory rather than solely to immediate analgesic response.

The higher point estimates at both metabolic extremes are compatible with the prespecified nonlinear hypothesis of distinct pathways at the two extremes. Among poor metabolizers, reduced conversion of hydrocodone to hydromorphone could contribute to inadequate analgesia, repeated clinical contact, opioid switching, or continued prescribing.^9,20,21^ Among ultrarapid metabolizers, accelerated active-metabolite formation could affect analgesic response, adverse effects, dose adjustment, or subsequent prescribing.^10,11^ These mechanisms remain provisional. Only the European-ancestry stratum contained reportable extreme-phenotype cells, with 180 poor and 60 ultrarapid metabolizers. Every other ancestry-by-phenotype cell at the extremes contained at most 20 participants or none. The ordinal substrate-tier trend and phenotype-by-tier interaction were nonsignificant, providing no evidence of a clear gradient according to CYP2D6 dependence. Healthcare-use adjustment attenuated the poor-metabolizer estimate from 1.48 to 1.33 and the ultrarapid-metabolizer estimate from 1.50 to 1.37, indicating that healthcare engagement or prescribing patterns may account for part of the association. Cox models remained directionally consistent. The PPI negative control was nonsignificant but imprecise. The null result is therefore an underpowered check rather than evidence against residual confounding. These analyses preserve the overall phenotype signal while limiting confidence in a single pharmacologic explanation.

Stratified estimates offer a preliminary read on whether the association holds across surgical groups (eTable 2 and eFigure 1 in Supplement). Every stratum-specific poor-metabolizer odds ratio exceeded 1.0, from 1.05 to 1.77 across all six region and procedure strata. The joint 3-degree-of-freedom phenotype test was nominally significant in four of the six strata, including the two largest, lumbar and combined procedures. Ultrarapid-metabolizer estimates were not directionally consistent across procedure strata. In the full cohort, the phenotype-by-procedure-type interaction was nominally significant (*P* = 0.033). That 6-degree-of-freedom omnibus test was unadjusted for the two interactions examined, and it appears driven by direction at the ultrarapid extreme rather than magnitude. It is best read as hypothesis-generating: the association may not be uniform across procedure types. The stratum-specific differences need replication before they are read as real.

The clinical implications are narrow and hypothesis-generating rather than practice-changing. The estimated absolute risk differences could become clinically meaningful if reproduced, especially for poor metabolizers receiving hydrocodone. Yet the cross-validated clinical model reached an AUROC of only 0.571, and adding CYP2D6 phenotype moved it to 0.572. Discrimination near 0.57 is close to chance, so the limit on clinical use is the clinical model itself, not the genotype increment alone. This distinction is important because clinically useful risk stratification requires not only statistical association but also adequate discrimination, validation, interpretability, and the ability to translate predicted risk into an actionable bedside framework.^22-24^ The findings do not support routine preoperative CYP2D6 testing or genotype-directed prescribing for prevention of persistent opioid use. Existing CYP2D6 results may still guide codeine or tramadol therapy under established CPIC recommendations.^9^ Clinical translation will require a reproducible medication-specific association and evidence that genotype information changes management beyond established risk factors.

Strengths include a spine-specific opioid-naive cohort with whole-genome sequencing, a recognized persistent-use definition, modeling aligned with a prespecified nonlinear hypothesis, adjustment for ancestry and clinical covariates, and complementary medication-specific, healthcare-use, negative-control, time-to-event, absolute-risk, and prediction analyses. Limitations include the retrospective design and use of prescription fills, which measure dispensing rather than consumption, pain severity, dependence, or misuse. Genotype-derived phenotype may not reflect functional CYP2D6 activity because inhibitor-related phenoconversion was unavailable. Opioid identity, dose, duration, adherence, multimodal analgesia, postoperative pain trajectories, refill indications, and reasons for continued use were incompletely captured. Poor- and ultrarapid-metabolizer groups were small, producing wide confidence intervals, and reportable extreme-phenotype analyses were confined to the European-ancestry stratum. Cervical, thoracic, and lumbar procedures were pooled, as were fusion and decompression. That pooling may have obscured procedure-specific effects, because postoperative complication, reoperation, and healthcare-utilization profiles can differ according to surgical approach even within lumbar decompression populations.^25^ Stratified estimates by anatomic region and procedure type are reported (eTable 2 in Supplement). Those strata carried as few as 100 events, one ultrarapid-metabolizer estimate could not be reported, and the interaction tests were exploratory, so procedure-specific effects remain incompletely resolved. Attenuation after healthcare-use adjustment and the imprecise negative control also leave residual confounding unresolved.

The immediate priority is independent replication in a larger, ancestry-diverse surgical cohort with adequate representation of poor and ultrarapid metabolizers. Future studies should capture opioid-specific exposure, dose, duration, CYP2D6 inhibitors, phenoconversion, multimodal analgesia, pain trajectory, actual consumption, and refill indications. Prescription fills measure dispensing, so prospective trials should track intake directly with electronic pill bottles or smart packaging, digital patient-reported-outcome platforms, and smartphone-based ecological momentary assessment. Artificial intelligence and digital twins could deliver pharmacogenomic decision support, and virtual reality rehabilitation could reduce opioid reliance. Neither application is tested.^26-28^ Prespecified analyses should test the nonlinear association and medication-specific interactions, particularly poor metabolizer status with hydrocodone, while reassessing absolute risk and incremental predictive value. A prospective genotype-guided intervention would be appropriate only after a reproducible association demonstrates clinically meaningful decision value.

## Conclusion

Among opioid-naive adults undergoing cervical, thoracic, or lumbar spine surgery, CYP2D6 metabolizer phenotype was associated with new persistent opioid use in a significant global analysis, with higher risk estimates among poor and ultrarapid metabolizers and a near-null estimate among intermediate metabolizers. The estimated absolute risk differences at the metabolic extremes may be clinically relevant, although phenotype-specific estimates were imprecise and sensitivity analyses indicated potential healthcare-related contributions. These findings support medication-specific, procedure-specific, ancestry-diverse replication before CYP2D6 testing is considered for prevention of persistent postoperative opioid use.

## Supplemental Material

Supplemental Material - CYP2D6 Metabolizer Phenotype and New Persistent Opioid Use After Spine Surgery Supplemental Material for CYP2D6 Metabolizer Phenotype and New Persistent Opioid Use After Spine Surgery by Samer G. Salman, Rohan A. Phadke, Krishna K. Anand, Joseph W. Guarnieri, Ethan Waisberg, James Rizkalla, Nathan J. Lee in Global Spine Journal

## Data Availability

This study used data from the All of Us Research Program Controlled Tier Dataset version 9 (C2025Q4R6), available to authorized users through the Researcher Workbench. Analysis code for cohort construction, variable derivation, statistical modeling, sensitivity analyses, and aggregate table generation is publicly available at https://github.com/samersalman/cyp2d6-spine-opioid-analysis and permanently archived on Zenodo (DOI:10.5281/zenodo.21616444) under the MIT License. All participant-level analyses were conducted within the secure Researcher Workbench. Participant-level data are not included in the repository or otherwise redistributed; reproduction requires authorized All of Us access and compliance with applicable data-use and dissemination policies.
