# Supplementary material for: CYP2D6 Metabolizer Phenotype and New Persistent Opioid Use After Spine Surgery
Source: Global Spine J. 2026 Sep 30:21925682261495118. Online ahead of print. doi: 10.1177/21925682261495118 (PMC13627458; doi:10.1177/21925682261495118)
Supplement: Supplementary file 1 [file sj-pdf-1-gsj-10.1177_21925682261495118.pdf]

Supplement

CYP2D6 Metabolizer Phenotype and New Persistent Opioid Use After Spine Surgery

Contents:

eTable 1. Perioperative opioid substrate tier and the poor-metabolizer association.
eTable 2. CYP2D6 metabolizer phenotype and new persistent opioid use, stratified by anatomic region and procedure type.
eFigure 1. Stratum-specific adjusted odds ratios for new persistent opioid use among poor and ultrarapid CYP2D6 metabolizers after spine surgery.

eTable 1. Perioperative opioid substrate tier and the poor-metabolizer association.

Perioperative opioid tier	N	Events	Poor metabolizers (n)	PM odds ratio (95% CI)	*p-value*
T3 codeine or tramadol (strong)	360	80	20	1.28 (0.44–3.72)	0.65
T2 hydrocodone (moderate)	680	120	60	2.15 (1.07–4.32)	0.032
T1 oxycodone (weak)	1,480	240	80	1.08 (0.58–2.02)	0.80
T0 non-substrate	260	40	≤20	Not estimated (thin cells)	n/a

Note: Tiers reflect the degree of CYP2D6 involvement in metabolizing the most CYP2D6-dependent opioid filled in the perioperative window.

eTable 2. CYP2D6 metabolizer phenotype and new persistent opioid use, stratified by anatomic region and procedure type.

Stratum	N	Events, n (%)	IM OR (95% CI)	PM OR (95% CI)	UM OR (95% CI)	Joint 3-df P
**Panel A. Anatomic region**						
Cervical	960	100 (9.9%)	0.99 (0.63–1.55)	1.38 (0.57–3.35)	1.81 (0.58–5.66)	0.081
Lumbar	2,280	240 (10.8%)	1.09 (0.82–1.44)	1.59 (0.94–2.68)	1.75 (0.91–3.36)	0.004
Other	560	120 (21.1%)	1.06 (0.68–1.65)	1.05 (0.46–2.40)	Not estimated (thin cells)	0.13
Phenotype x anatomic region interaction (full cohort): χ^2 = 12.04; df = 6; P = 0.061						
**Panel B. Procedure type**						
Fusion	1,080	140 (13.0%)	1.07 (0.74–1.56)	1.22 (0.60–2.48)	1.73 (0.69–4.37)	0.045
Decompression	1,300	120 (9.2%)	1.15 (0.77–1.71)	1.77 (0.86–3.61)	0.89 (0.28–2.82)	0.034
Both	1,420	200 (14.1%)	0.98 (0.70–1.35)	1.54 (0.83–2.85)	2.17 (0.98–4.80)	0.006
Phenotype x procedure type interaction (full cohort): χ^2 = 13.69; df = 6; P = 0.033						

Abbreviations: CI, confidence interval; CYP2D6, cytochrome P450 2D6; IM, intermediate metabolizer; NM, normal metabolizer; OR, odds ratio; PM, poor metabolizer; UM, ultrarapid metabolizer. Other comprises thoracic, multilevel (procedures spanning more than one spinal region), and unassignable operative levels.

Odds ratios are from Firth-penalized logistic regression fitted within each stratum, with normal metabolizers as the reference group. Models adjusted for age, sex, pre-index body mass index, baseline back pain, depression, anxiety, substance-use disorder, Charlson comorbidity index, and ancestry principal components 1 through 10. Panel A additionally retained procedure stratum as a covariate, because procedure type varies within an anatomic region; Panel B did not, because procedure type defines its strata. The joint 3-degree-of-freedom P value is a penalized likelihood-ratio test of the four-level phenotype within that stratum. The interaction rows are penalized likelihood-ratio comparisons of the interaction model against the main-effects model in the full cohort (N=3,820, 460 events); they are 6-degree-of-freedom omnibus tests and were not adjusted for the two tests performed, so they are exploratory and hypothesis-generating.

A stratum-by-phenotype cell was modelled only where the stratum contained at least 150 participants and at least 30 events and the phenotype group contained at least 20 participants; one cell did not meet this rule and is reported as not estimated. Phenotype-group sizes were, in Panel A, cervical IM 360, PM 60, UM 20; lumbar IM 840, PM 120, UM 80; other IM 220, PM 40, UM ≤20; and in Panel B, fusion IM 420, PM 80, UM 40; decompression IM 500, PM 80, UM 40; both IM 500, PM 80, UM 40. Counts are rounded to the nearest 20, and cells with 20 or fewer participants are suppressed, in accordance with All of Us Research Program disclosure requirements. The anatomic-region counts therefore sum to 3,800 against a primary analytic N of 3,820; the region event counts sum exactly to the 460 observed events, so the shortfall is in the denominators only. Event percentages for anatomic region were calculated before disclosure rounding. Those for procedure type were calculated from the rounded counts shown. Percentages therefore may not correspond exactly to the reported counts.

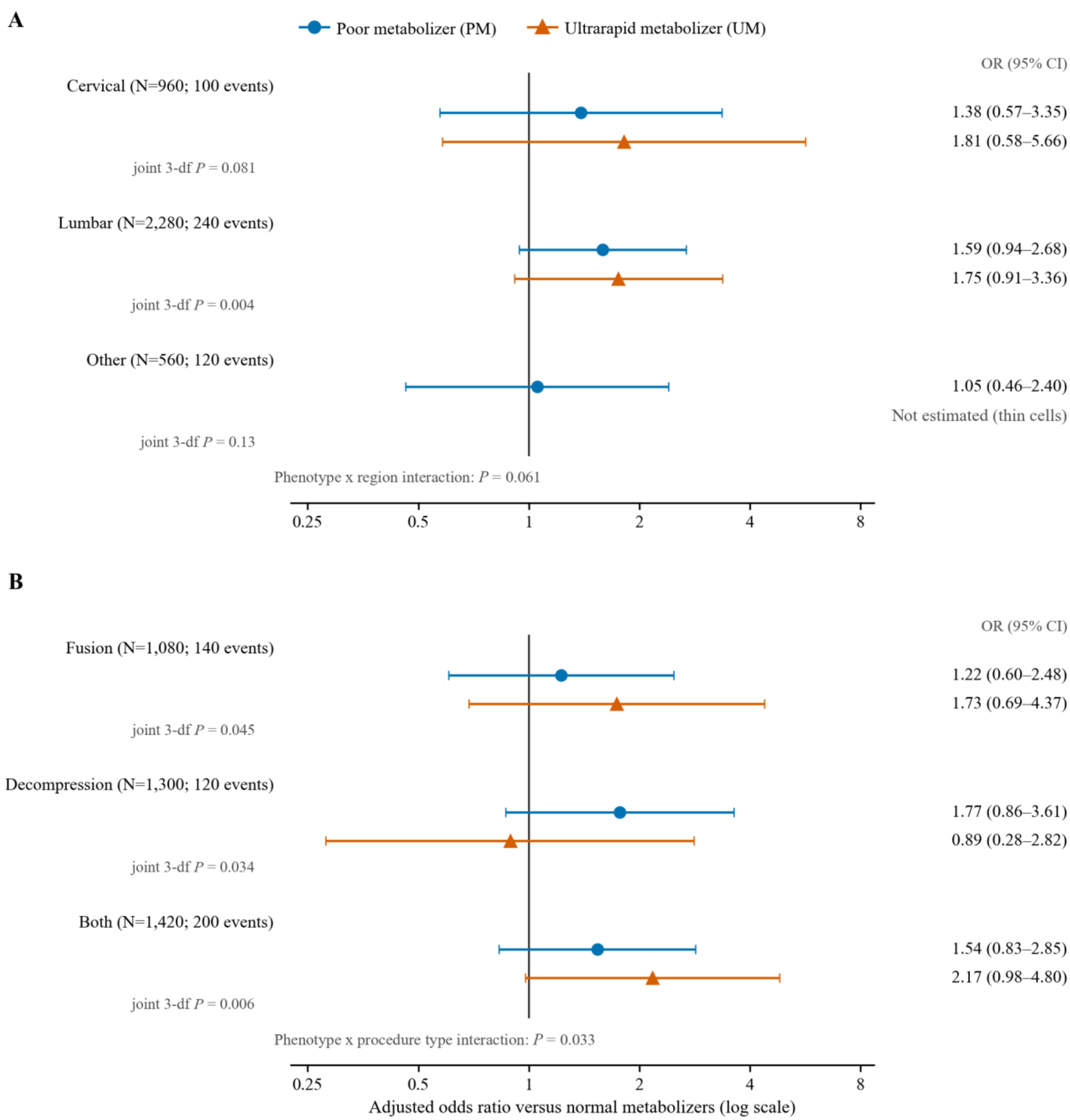

eFigure 1. Stratum-specific adjusted odds ratios for new persistent opioid use among poor and ultrarapid CYP2D6 metabolizers after spine surgery. Panel A shows estimates within anatomic region strata: cervical, lumbar, and other, where other comprises thoracic, multilevel (procedures spanning more than one spinal region), and unassignable operative levels. Panel B shows estimates within procedure strata: fusion, decompression, and both, the combined-procedure group. Markers are adjusted odds ratios (OR) and horizontal lines are 95% confidence intervals, plotted on a logarithmic axis with the solid vertical line at the null value of 1.00. No confidence interval is truncated. Each stratum row is annotated with its participant count, its event count, and the joint 3-degree-of-freedom penalized likelihood-ratio test for the 4-level CYP2D6 phenotype within that stratum.

Estimates come from Firth-penalized logistic regression fitted separately within each stratum, with normal metabolizers as the reference group. Models adjusted for age, sex, pre-index body mass index, baseline back pain, depression, anxiety, substance-use disorder, Charlson comorbidity index, and ancestry principal components 1 through 10. Panel A additionally retained procedure stratum as a covariate, because procedure type still varies within an anatomic region. Panel B dropped that covariate, because procedure type defines the strata themselves.

Intermediate-metabolizer estimates were near the null in every stratum, ranging from 0.98 to 1.15, and are therefore not plotted. Complete estimates for all three phenotype contrasts are reported in eTable 2.

In the full cohort, the phenotype-by-region interaction was nonsignificant (P = 0.061) and the phenotype-by-procedure-type interaction was nominally significant (P = 0.033). Both come from penalized likelihood-ratio comparisons of the interaction model against the main-effects model and are 6-degree-of-freedom omnibus tests. These analyses were exploratory and were not adjusted for the two tests performed, so the procedure-type result is hypothesis-generating. It indicates that the phenotype association may not be uniform across surgical groups rather than establishing where it differs.

Participant and event counts are rounded to the nearest 20 in accordance with All of Us Research Program disclosure policy, so the Panel A region counts sum to 3,800 rather than the analytic total of 3,820; the region event counts sum exactly to the 460 observed events, so the shortfall is in the denominators only. The ultrarapid-metabolizer estimate in the other-region stratum was not estimated because of thin cells and is shown without a marker.
